# Factors associated with stress-related symptoms among people with epilepsy in Ethiopia, a cross-sectional study

**DOI:** 10.3389/fneur.2023.1218448

**Published:** 2023-07-31

**Authors:** Jemal Seid, Birhane Gebrehiwot, Fantahun Andualem, Abenet Kassaye, Jemal Mohammed, Mulugeta Akele

**Affiliations:** ^1^Department of Psychiatric Nursing, College of Health Sciences, Mekelle University, Mekelle, Ethiopia; ^2^Department of Psychiatric Nursing, College of Health Sciences, Adigrat University, Adigrat, Ethiopia; ^3^Department of Medicine, Dessie Comprehensive Specialized Hospital, Dessie, Ethiopia; ^4^Department of Psychiatry, College of Medicine and Health Science, Mizan-Tepi University, Mizan, Ethiopia

**Keywords:** comorbidity, epilepsy, Mekelle, people with epilepsy, stress-control

## Abstract

**Introduction:**

Stress is a common psychological problem present in people with epilepsy and has a serious impact on the health-related satisfaction of people with epilepsy and their cohabiters. This study aimed to assess the magnitude and related factors of stress.

**Methods:**

A hospital-based cross-sectional study was carried out among 301 systematically chosen people with epilepsy. The seven stress-related items of the Depression, Anxiety, and Stress -21 questionnaire was used to measure stress. Data were entered using Epi Info and analyzed by SPSS version 25. Predictors with a *p*-value < 0.20 in the bivariate logistic regression were transferred into the multivariate model. A *p-*value of less than 0.05 was viewed as statistically significant.

**Result:**

The prevalence of stress symptoms in this study was 23.9%. Daily labor occupational status with Adjusted Odds ratio [(AOR) = 0.042, 95% CI: 0.004, 0.469], onset of illness at the age of 18 years and above (AOR = 0.188, 95% CI: 0.046, 0.771), perceived stigma (AOR = 3.320, 95% CI: 1.345, 8.200), the presence of anxiety symptoms (AOR = 8.275, 95% CI: 3.345, 20.471), and belief that the condition is untreatable (AOR = 6.360, 95% CI: 1.647, 24.562) were significantly associated factors.

**Conclusion:**

The occurrence of stress was high, and it reinforced that there is a requisite for the identification and handling of stress-related symptoms among people with epilepsy.

## 1. Introduction

Epilepsy is a treatable noninfectious disease that can affect people at any age (1). A global survey carried out by the WHO showed that 50 million people are affected by epilepsy (2) and 90% of them are found in low-income countries (3). Many studies have shown that epileptic patients significantly experience higher levels of psychiatric and psychological comorbidity (3–6). Various studies have shown that the common comorbid psychiatric disorders reported by persons with epilepsy are anxiety, depression, stress, attention deficit disorder, and psychosis (5, 7, 8).

Epilepsy has several emotional consequences that reduce the health-related satisfaction of the person with epilepsy, their household, and their community in multiple ways (9, 10). People with epilepsy (PWE) report stress as the usual trigger for their seizures (11–16) and some also believe that it is a cause for their epilepsy (6, 17). Stress among PWEs is a common psychological symptom because they experience unpredictable seizures, underemployment, stigma, social discrimination, and antiepileptic drug (AED) side effects (9, 13, 18). Stress is the individual’s physiological and/or behavioral response to a certain threatening activity/event (6, 12).

Stress occurs when environmental demands exceed an individual’s adaptive ability, resulting in emotional and biological changes (13, 19). Due to the onset of provoking and unusual symptoms, individuals with epilepsy often face a lack of social support, low self-esteem, and discrimination. As a result, they also develop stress, sadness, and anxiety (9, 20). During their life, PWE can undergo acute stress, in which stress occurs for a brief time, and chronic stress, in which stress occurs constantly over a prolonged period (13). A report carried out in Thailand showed that 76.4% of the patients experienced medium or high levels of stress, and epilepsy was reported as the root cause for their stress (50.2%) (18).

Another study in Pakistan stated that 70% of the respondents experienced psychological distress; lack of occupation, the presence of an underlying disabling condition, and the severity of epilepsy were independent variables (20). A study conducted in the Netherlands among children with epilepsy showed that 62% of them develop stress, and the number of antiepileptic drugs and sleep quality are the determining factors (14). A study conducted in Ethiopia involving parents of children with epilepsy found that 27.1% of participants were experienced psychological distress. Factors such as being a parent, having low levels of social support, and experiencing stigma were identified as independent predictors of stress (21).

A person with psychological disturbance may display other psychiatric symptoms such as nervousness, confused feelings and perception, temper, hopelessness, and hostility (6, 18). Stress is universal but there is variability in its source, strength, and treatability (6, 22). More recent studies have shown that cognitive-behavioral therapy has been associated with a substantial reduction of stress among PWE (23).

Stress-reduction behavioral interventions designed to reduce the physical and behavioral effects of stress can be effective (24). Another study also stated that psychoactive medications are often prescribed in the context of stress-related disturbances (6, 25). For PWE, stress is a principal component that precipitates seizures, decreases AED adherence, and reduces health-related personal satisfaction (9–12, 14). Stress has become a common comorbid problem for people with epilepsy. Providing good information about the comorbidity of stress with epilepsy is one of the main ways to promote healthy behavior among people with epilepsy (6, 26). This indicates that there is a need to identify the factors that trigger stress symptoms.

Health professionals should also be helped to design strategies against the possible risk factors of stress among epileptic outpatients. The results of this study will provide some information for policymakers to design and integrate a comorbid screening approach with the general health service to minimize comorbidity. The study could also provide information for MOH, NGOs, health extension workers, and other responsible bodies to develop a campaign that could increase awareness of the comorbidity of mental illness with chronic health problems including epilepsy. Finally, the study could offer a direction to establish holistic treatment approaches in addition to the existing neurological services. These research findings were used as a baseline data source for other related studies. There have been a limited number of studies conducted on this issue globally, and none conducted in Ethiopia. Since there is a paucity of information about stress and its associated factors among people with epilepsy, this study aims to assess the magnitude of stress and its associated factors to reduce the paucity of information.

## 2. Methods and material

### 2.1. Study areas, design, and period

An institutional-based cross-sectional study was conducted from April 15/2019 to May 30/2019 at the neurologic outpatient department of Ayder Comprehensives Specialized Hospital (ACSH) and Mekelle General Hospital (MGH) in the northern part of Ethiopia.

### 2.2. Source population and study population

All outpatients who were receiving neurologic services in the neurology outpatient department of ACSH & MGH were the source of population. The study population included patients with epilepsy who were newly diagnosed and/or receiving follow-up care at ACSH and MGH during the study period. Based on their consent and assent, those newly diagnosed epileptic patients and those receiving regular follow-up treatment who were 12 years of age or older were asked to participate in study. However, PWE who were critically ill, unable to speak and hear, or were aged from 12 to17 years without supervision were excluded from the study.

### 2.3. Sample size and sampling procedure

The sample size of 301 was determined based on the formula for a single population proportion by assuming the prevalence of stress among epileptic patients 50% confidence level 95%, margin of error 5, and 5% for non-response rate. Finally, the study participants were proportionally allocated. A systematic random sampling method was applied to select study subjects from both settings. The *k* value was computed by dividing the total number of people with epilepsy by the total study subject (1,103/301 ≈ 3). The data was gathered over a six-week duration; the total PWE followed in 1 month was gained by calculating the average of the preceding year. The desired sample size was equivalently assigned to each hospital. Finally, to select the patients for data collection, every third person visiting the respected hospital was chosen by the data collectors.

### 2.4. Data collection tool and data collection procedure

Two trained BSc degree psychiatric professionals who work in a clinical setting gatherd the information by applying an interviewer-administered pre-tested questionnaire. The questionnaire had sociodemographic features (such as age, sex, marital status, and educational level) and questions that assessed the features allied with stress. Stress was evaluated by the seven stress-related items of the depression, anxiety, and stress scale-21 (DASS-21). DASS 21 is a 21-item tool developed to evaluate the level of depression, anxiety, and stress symptoms. However, for this study, we selected the modified form of DASS-21 that contains seven items with a great emphasis on evaluating the harshness of the core indicators of stress symptoms only. This instrument is cross-culturally valid in China with a Cronbach alpha of = 0.86 and, when adopted in Ethiopia, is reliable with a Cronbach’s alpha of 0.86 (27). An outcome ≥10 is considered as experiencing moderate to severe stress.

To assess depression and anxiety, a patient health questionnaire-9 (PHQ-9) and generalized anxiety disorder-7 (GAD-7) were used. PHQ-9 is a broadly used tool for assessing depression. It is a reliable and valid measure of depression in various cultural groups with a Cronbach’s α range from 0.84 to 0.915 in different nations (28, 29). It was also formalized in Afaan Oromo Cronbach’s alpha, 0.84 (30). A score >10 is considered as having a depression (31).

The GAD-7 is a commonly used tool for screening anxiety symptoms over the past 2 weeks. It consists of seven items with four Likert scale responses. It has been cross-culturally validated with a Cronbach’s α of 0.915, indicating good internal consistency (32). A score above nine is considered as having moderate to severe anxiety disorder (31). Patients who scored ≥10 on the PHQ-9, GAD-7, and ≥15 on the stress assessment of DASS-21 were linked as volunteers to the psychiatry outpatient department for further assessment and management. The perceived stigma was assessed by applying the Kilifi Stigma Scale of Epilepsy. It originated and was confirmed in Kilifi, Kenya, with high internal uniformity and Cronbach’s 𝛼 of 0.91 (33). It is reliable in an Ethiopian context (34, 35). It has 15 items with a three-point Likert scoring system. The lower limit score was 0 and the uppermost score was 30. The 66th percentile was a cut-of point to categorize stigma. Scores above the 66th percentile indicated greater stigmatization feeling (34, 35).

The Oslo 3-item social support scale assessed the level of social support. It is a 3-item instrument frequently used to assess social support. The internal consistency of the Oslo 3-item social support scale could be viewed as acceptable with α = 0.640 (36, 37). It judges the simplicity of receiving aid from neighbors, the number of people the subjects can count on when there are serious problems, and the level of concern people show in what the subject is doing. The range of the tool is 3–14. The scores are understood as 3–8 (poor social support), 9–11 (moderate social support), and 12–14 (strong social support) (38, 39). To assess substance use, an adapted form of the ASSIST formulated by the World Health Organization (WHO) was applied. The average reliability test coefficients ASSIST ranged from a Cronbach’s 𝛼 of 0.58–0.90, which means it is good to excellent in reliability (40–42).

### 2.5. Data quality assurance

To retain the quality of the study‘s data, the questionnaires were transformed into Tigrigna (local language) by an expert Tigrigna speaker who had skill and knowledge in psychological illness. The translations were back-translated to English by a senior. Two weeks before the final data collection, a pre-test was performed on 5% of the total sample of study subjects in Quiha General Hospital to determine easiness, feasibility, and applicability instruments, to estimate the total time required for responding, and to recognize barriers that may be experienced throughout data collection. The sample in the pre-test was excluded from the final sample of the research work. Additionally, the principal investigator gave one-day face-to-face training for interviewers on the approaches of data collection (from initial communication up to final result scoring). The collected pieces of information were assured for completeness every day.

### 2.6. Data analysis procedure

Data were entered and checked by means of Epi-info version 4.4.3.1 and transferred to Statistical Package for Social Sciences version 25 for additional analysis. Descriptive statistical analysis was carried out to indicate the frequencies and percentages of the variables. Binary logistic regression and adjusted odd ratio with a 95% confidence interval were employed to determine the associated factors of stress. All factors with a *p-*value < 0.20 in the bivariate logistic regression were directly figured into the multivariate logistic regression. Lastly, all *p*-values less than 0.05 were considered statistically significant.

### 2.7. Ethical consideration

Ethical approval was obtained from the ethical review board of Mekelle University College of Health Science. A written agreement form was taken from each individual. For those aged 12 to17, written assent was taken from their families that accompanied the patient. All collected data were used for this paper only. An information sheet was attached to each inquiry form to provide study details. This form indicated that involvement is voluntary and participants have the right to retire from completing the questionnaire at any time. Participants were assured that if they wished to refuse to participate, their care or dignity would not be compromised in any way since there was no association between participation and health or treatment outcome. Participants were also informed that there was no expectation of supplementary treatment or any benefits for them associated with participating in the study but, based on their consent, those who scored above 9 on the PHQ-9 and -GAD-7 and/or above 14 on the stress assessment of DASS-21 were linked to the psychiatric outpatient department of the respected hospital for better screening and intervention.

## 3. Results

### 3.1. Sociodemographic characteristics of the study subject

A total of 301 patients with epilepsy were employed in the study. The overall response rate was 100%. All the responders were in the age range of 13–65 years with a mean age of 31.52 years. The median and mode ages of the participants were 28.0 and 24, respectively.

Out of the total participants, 162 (53.8%) were male, 185 (61.5%) lived in an urban area, and geographically 274 (91.0%) were Tigran in ethnicity. A majority of the participants 245 (81.4%) were orthodox in religion, 103 (34.2%) were unable to read or write, 146 (47.5%) were single, 144 (47.8%) had no monthly income, and 173 (57.5%) had moderate public support (Table 1).

**Table 1:** Distribution of study subjects by socio-demographic factors (*n* = 301) in Ethiopia.

	Variables	Frequency	Percentage
Age	12–17 years	38	12.6
	18–24 years	80	26.6
	25–34 years	72	23.9
	35–44 years	57	19.0
	>44 years	54	17.9
Sex	Male	162	53.8
	Female	139	46.2
Residency	Urban	185	61.5
	Rural	116	38.5
Ethnicity	Tigray	274	91.0
	Amhara	11	3.7
	Afar	15	5.0
	Other	1	0.3
Religion	Orthodox	245	81.4
	Catholic	4	1.3
	Protestant	4	1.3
	Muslim	48	16.0
Educational status	Unable to read and write	103	34.2
	Primary 1–8	91	30.3
	Secondary 9–12	48	15.9
	Techniques	8	2.7
	Diploma	7	2.3
	First degree and above	44	14.6
Marital status	Married	106	35.2
	Single	146	48.5
	Divorced	22	7.3
	Widowed	25	8.3
	Other	2	0.7
Employment	No	39	13.0
	Student	76	25.2
	Farmer	32	10.6
	Housewife	63	21.0
	Government	16	5.3
	Private	31	10.3
	Others	26	8.6
	Merchant	18	6.0
Monthly income in Birr	No	144	47.8
	301–600	24	8.0
	<300	24	8.0
	601–1,000	25	8.3
	>1,000	84	27.9
Social support	Low	71	23.6
	Moderate	173	57.5
	High	57	18.9

### 3.2. The description of study subject by clinical factors

From 301 study subjects, 115 (38.2%) started the illness at 18 years of age and above, 127 (42.2%) had seizure-related physical trauma. 169 (56.1%) of the participants reported that they undergo ≤2 seizures in a month, and 85 (28.2%) experienced 3–5 seizures per month.

A majority of the study participants 223 (74.1%) were on a single-treatment regime, 87 (28.9%) had used the treatment service for >11 years, and 271 (90.0%) had better enhancement with medication, and 70 (23.3%), 92 (30.6%), and 86 (28.6%) reported depression symptoms, feelings of stigmatization, and anxiety symptoms, respectively (Table 2).

**Table 2:** Distribution of study subjects by clinically related factors of peoples with epilepsy (*n* = 301) in Ethiopia.

	Variables	Frequency	Percentage
Age of onset	<6 years	35	11.6
	6–11 years	78	25.9
	12–17 years	73	24.3
	18 and above years	115	38.2
Number of seizures	≤2 per month	169	56.1
	3–5 per month	85	28.2
	6–10 per 6 months	38	12.6
	≥11 per year	9	3.0
Seizures-related physical trauma	No	174	57.8
	Yes	127	42.2
Number of AEDs	Monotherapy	223	74.1
	Polytherapy	78	25.9
Length of time with medication	0–11 month	32	10.6
	Less than 2 years	45	15.0
	2–5 years	82	27.2
	6–10 years	55	18.3
	11 or more years	87	28.9
Improvement with medication	No	30	10.0
	Yes	271	90.0
Medication-related side effects	No	217	72.1
	Yes	84	27.9
Lifetime substance use history	No	232	77.1
	Yes	69	22.9
Substance use history in the past 3 months	No	268	89.0
	Yes	33	11.0
Presence of chronic medical illness	No	256	85.0
	Yes	45	15.0
Depression	No	231	76.7
	Yes	70	23.3
Perceived stigma	No	209	69.4
	Yes	92	30.6
Anxiety	No	215	71.4
	Yes	86	28.6
History of physical abused	No	287	95.3
	Yes	14	4.7
History of sexual abused	No	299	99.3
	Yes	2	0.7
Suicidal attempt history	No	290	96.3
	Yes	11	3.7
Presence of suicidal wish	No	244	81.1
	Yes	57	18.9

### 3.3. The description of a study subject by contagion belief and causal belief of epilepsy

Regarding the cause of epilepsy, 79 (26.2%) believed that epilepsy is a result of walking around garbage, dumps, ashes, or walking along a river; 79 (26.2%) did not recognize the cause. Out of the total respondents, 179 (59.5%) believed that epilepsy is a psychiatric disorder and 270 (89.7) believed that epilepsy is manageable (Table 3).

**Table 3:** Distribution of study subjects’ beliefs on epilepsy (*n* = 301) in Ethiopia.

	Variables	Frequency	Percentage
Cause of epilepsy	I do not know	79	26.2
	Spiritual possession	41	13.6
	Evil eye	20	6.6
	Family history	25	8.3
	Pathogens	9	3.0
	Sinful act	31	10.3
	Walks around garbage, dumps, ashes, walking along a river	79	26.2
Contagious	No	265	88.0
	Yes	36	12.0
Heritable	No	246	81.7
	Yes	55	18.3
Mental illness	No	122	40.5
	Yes	179	59.5
Treatable	No	31	10.3
	Yes	270	89.7

### 3.4. The prevalence of stress

**Figure 1:**
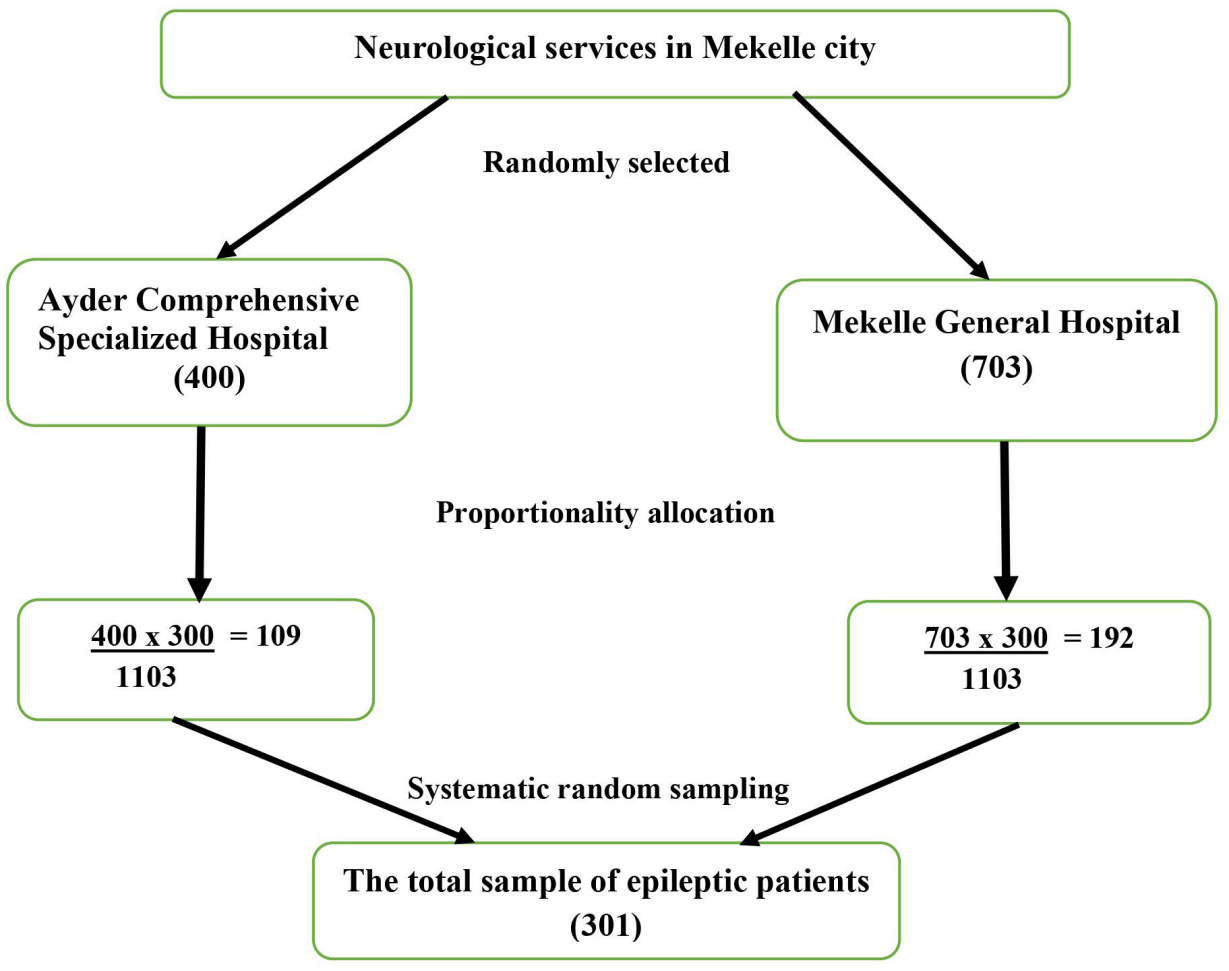
Sample size proportionality for the study of factors associated with stress disorder symptoms among PWE in Ethiopia.

Overall, the prevalence of stress was found to be 23.9% with a 95% CI [19.0–29.0] (Figure 1).

### 3.5. Factors associated with stress

Multivariate analyses were performed between stress and response variables of sociodemographic variables (Age, educational status, Marital status, employment, and social support), clinical factors (age of onset, treatment duration, presence of chronic medical illness, sense of stigmatization, depression symptoms, anxiety symptoms, and suicidal thoughts), and patient belief factors (treatability belief of epilepsy).

Accordingly, occupational status of being a daily laborer (AOR = 0.042, 95% CI: 0.004, 0.469), starting the illness at the age of 18 years or above (AOR = 0.188, 95% CI: 0.046, 0.771), perceived stigma (AOR = 3.320, 95% CI: 1.345, 8.200), the presence of anxiety symptoms (AOR = 8.275, 95% CI: 3.345, 20.471), and belief that the condition is untreatable (AOR = 6.360, 95% CI: 1.647, 24.562) were significantly associated with stress symptoms (Table 4).

**Table 4:** Factors associated with stress symptoms (bivariate and multivariate analysis) among PWE in Ethiopia.

	Stress		Bivariate analysis		Multivariate analysis	
Variables	No	Yes	*p*-value	COR (95%, CI)	AOR (95%, CI)	*p*-value
**Age**						
12–17 years	29	9	0.284	1.784 (0.618, 5.149)	0.570 (0.068, 4.813)	0.606
18–24 years	59	21	0.119	2.047 (0.831, 5.039)	1.365 (0.210, 8.873)	0.744
25–34 years	52	20	0.088	2.212 (0.889, 5.499)	0.670 (0.145, 3.089)	0.607
35–44 years	43	14	0.202	1.872 (0.715, 4.904)	1.117 (0.254, 4.910)	0.883
>45 years	46	8	1			
**Marital status**						
Married	81	25	1			
Single	104	42	0.359	1.308 (0.737, 2.323)	2.728 (0.758, 9.8160)	0.125
Divorced	18	4	0.583	0.720 (0.223, 2.326)	1.009 (0.168, 6.078)	0.992
Widowed	26	1	0.056	0.135 (0.017, 1.049)	0.049 (0.001, 1.616)	0.091
**Employment**						
No	24	15	0.669	0.781 (0.252, 2.422)	0.165 (0.024, 1.124)	0.066
Student	61	15	0.034	0.307 (0.104, 0.912)	0.489 (0.066, 3.615)	0.483
Farmer	24	8	0.162	0.417 (0.122, 1.421)	1.045 (0.133, 8.233)	0.967
Housewife	49	14	0.067	0.357 (0.118, 1.076)	0.815 (0.133, 4.981)	0.825
Governmental	13	3	0.119	0.288 (0.060, 1.376)	0.898 (0.066, 12.165)	0.936
Private	24	7	0.115	0.365 (0.104, 1.278)	2.498 (0.295, 21.130)	0.401
Others/day labor	10	8	0.010	0.104 (0.019, 0.579)	0.042 (0.004, 0.469)	0.010**
Merchant	24	2	1			
**Social support**						
Low	51	20	0.100	2.092 (0.868, 5.042)	1.718 (0.461, 6.399)	0.420
Moderate	130	43	0.160	1.764 (0.800, 3.891)	1.653 (0.553, 4.938)	0.368
High	48	9	1			
**Age of onset**						
<6 years	20	15	1			
6–11 years	54	24	0.001	5.885 (2.431, 14.243)	0.748 (0.230, 2.437)	0.630
12–17 years	53	20	0.006	3.487 (1.645, 7.391)	0.376 (0.108, 1.312)	0.125
18 & above years	102	13	0.000	2.961 (1.367, 6.415)	0.188 (0.046, 0.771)	0.020**
**Treatment duration**						
0–11 month	27	5	1			
less than 2 years	39	6	0.777	0.831 (0.230, 3.001)	2.666 (0.464, 15.314)	0.272
2–5 years	70	12	0.894	0.926 (0.298, 2.877)	0.941 (0.188, 4.712)	0.941
6–10 years	36	19	0.063	2.850 (0.945, 8.599)	4.597 (0.907, 23.311)	0.066
11 or more years	57	30	0.052	2.842 (0.993, 8.135)	2.500 (0.505, 12.362)	0.261
**Adherence with medication**						
No	23	7	0.937	0.965 (0.396, 2.351)	1.979 (0.824, 4.752)	0.127
Yes	206	65	1			
**Seizure related trauma**						
No	139	35	1			
Yes	90	37	0.071	1.633 (0.958, 2.782)	0.827 (0.364, 1.882)	0.651
**Chronic medical illness**						
No	190	66	1			
Yes	39	6	0.077	0.443 (0.179, 1.094)	0.443 (0.092, 2.126)	0.309
**Depression**						
No	188	43	1			
Yes	41	29	0.000	3.092 (1.732, 5.521)	2.113 (0.851, 5.246)	0.107
**Perceived stigma**						
No	179	30	1			
Yes	50	42	0.000	5.012 (2.852, 8.807)	3.320 (1.345, 8.200)	0.009**^a^
**Anxiety**						
No	189	26	1			
Yes	40	46	0.000	8.360 (4.636, 15.075)	8.275 (3.345, 20.471)	0.000**^a^
**Treatable belief**						
No	19	12	0.046	2.211 (1.016, 4.811)	6.360 (1.647, 24.562)	0.007**^a^
Yes	210	60	1			

^*^Significantly associated at *p* < 0.2, ^**^statistically significant at *p* < 0.05.

a Statistically significant at *p* < 0.01.

*p*-value of Hosmer and Lemeshow goodness of fit test was = 0.625.

## 4. Discussion

The findings of the current study agree with another study conducted in Ethiopia among parents of children with epilepsy with a reported prevalence of 27.1% (21). The prevalence of stress in this study (23.9%) is higher when compared with other studies conducted in Germany 18% (43) and in Ethiopia among antenatal women and patients with HIV/AIDS (11.6 and 7.4%, respectively) (44, 45). In contrast, the magnitude of stress in this study (23.9%) is lower than a study conducted in the U.S. at 74% (46), in Pakistan (70%) (20), in Thailand at 76.4 and 77% (18, 47), in Nepal at 75.7% (48), in Nigeria at 69.1% (49), in India at 35% (50), in Egypt among medical students at 62.4% (51), and in Ethiopia conducted on college students at 63.7% (52). This latter result might be due to the situations of the respondent’s academic purpose, and separation from family contributing to the highest prevalence of stress. Generally, the discrepancy might be a result of the difference in assessment tools, geographical areas, sample size, the nature of the problem on the study subject, study setting, and cultures of the study subject. Concerning the associated factors, those who were involved in daily labor occupation were associated with stress with (AOR = 0.042, 95% CI: 0.004, 0.469) in which the daily laborers were 95.8% less likely to develop stress than those who worked on their business. One possible explanation is that individuals who rely on daily income may have fewer additional concerns that contribute to stress.

The likelihood of experiencing stress was significantly lower (AOR = 0. 88, 95% CI: 0. 46, 0. 71) among individuals who developed the illness at the age of 18 years or above compared to those who developed it before the age of 6 years. This result is also supported by many other studies conducted in India (50) and Germany (43). One possible explanation is that in this age group their interaction with the environment increased and they became more sensitive to eliciting a stress response.

Findings also proved that people with epilepsy who experienced stigma were 3 times (AOR = 3.320, 95% CI: 1.345, 8.200) more likely to have stress when compared with those who were not stigmatized. Other studies in Ethiopia among caregivers of PWE (21) and people with HIV/AIDS (45) also support this finding. This might be partly elucidated by the fact that stigma causes a multidimensional effect on the psychology of respondents, causing them to think more about isolation, loss of social support, and decreased social relationships.

In this study, anxiety was found to be another determinant factor for stress symptoms. Participants who experienced anxiety symptoms were eight times (AOR = 8.275, 95% CI: 3.345, 20.471) more likely to have stress than those who did not. This finding is supported by a study conducted at the University of Miami (52, 53) and in Korea (54), which suggests that individuals with epilepsy who experience symptoms of anxiety may struggle to engage in daily activities and community participation equally. They might face difficulties with socioeconomic stressors such as school dropout, joblessness, immiseration, and economic reliance on others. All these factors contribute to the development of psychological disturbances such as stress.

The current study findings also reveal that people with epilepsy who believe that epilepsy is untreatable were six times (AOR = 6.360, 95% CI: 1.647, 24.562) more likely to develop stress when compared to those who did not. One possible reason for this is that individuals with untreatable beliefs may limit their social interactions and experience reduced self-esteem due to their psychiatric illness; then, they also limit social activities such as marriage and education and may perceive themselves as unsuccessful, dependent, and handicapped. So, they think more and more about the above conditions. Because of this, they may develop stress.

### 4.1. Limitation of the study

It is important to consider potential recall and response biases that may have occurred during the completion of the questionnaire. Additionally, some independent variables, such as medication adherence, physical and sexual abuse, and the presence of suicidal thoughts, were assessed using single questions, which may have led to some patients responding inappropriately. Even though many studies (55–58) report high psychological consequences of epilepsy on family members, the current study was limited to people with epilepsy only.

Because of the cross-sectional study design, the research did not demonstrate any cause and effect association between the possible determinate factors and the outcome of interest.

### 4.2. Conclusion

The prevalence of stress among PWE was higher than the overall residents and was significantly associated with those who believed that epilepsy is untreatable and who have anxiety and stigma. In this study, factors such as daily labor occupation status and people who started the illness at the age of 18 years or above had a preventive association. Clinicians and healthcare professionals should be aware of an augmented risk of emerging stress-related symptoms. Therefore, systemic and holistic methods of assessment are needed to minimize the magnitude of stress among PWE.

## Data availability statement

The raw data supporting the conclusions of this article will be made available by the authors, without undue reservation.

## Ethics statement

This study was conducted after receiving ethical approval from Mekelle University, College of Health Science Office of Health Research Ethics Review Committee (HRERC) with the reference number Notification of Expedited Approval ERC 1301/2019. Written informed consent to participate in this study was provided by the participants’ legal guardian/next of kin.

## Author contributions

JS was the study’s primary investigator and made significant contributions to the conception, selecting the design, supervising and handling data collection, and analyzing and interpreting data. BG, FA, AK, JM, and MA were involved in drafting and critically revising the manuscript. All authors agreed to be accountable for all aspects of the work in ensuring that questions related to the accuracy or integrity of any part of the work are appropriately investigated and resolved. All authors contributed to the article and approved the submitted version.

## Funding

This work was supported by Mekelle University, College of Health Science. The funders had no role in the preparation of the manuscript or decision to publish.

## Conflict of interest

The authors declare that the research was conducted in the absence of any commercial or financial relationships that could be construed as a potential conflict of interest.

## Publisher’s note

All claims expressed in this article are solely those of the authors and do not necessarily represent those of their affiliated organizations, or those of the publisher, the editors and the reviewers. Any product that may be evaluated in this article, or claim that may be made by its manufacturer, is not guaranteed or endorsed by the publisher.
